# Dicylopenta­dien­yl[4-(4-vinyl­benz­yloxy)pyridine-2,6-dicarboxyl­ato]titanium(IV) monohydrate

**DOI:** 10.1107/S1600536810021148

**Published:** 2010-06-16

**Authors:** Fanghua Zhu, Yuancheng Qin, Jiehong Lei, Lin Zhang, Qiang Yin

**Affiliations:** a Research Center of Laser Fusion, China Academy of Engineering Physics, Mianyang 621900, People’s Republic of China; b College of Chemistry, Sichuan University, Chengdu 610064, People’s Republic of China

## Abstract

The title compound, [Ti(C_5_H_5_)_2_(C_16_H_11_NO_5_)]·H_2_O, exhibits a titanocene unit coordinated to a styrene-substituted pyridine-2,6-dicarboxyl­ate ligand synthesized for use as a monomer for polymerization or copolymerization yielding metallocene-containing polymers. The compound crystallized as a monohydrate and the solvent water mol­ecule forms strong O—H⋯O hydrogen bonds with the carboxyl­ate O atoms of the Ti complex, which play an important role in the connection of adjacent mol­ecules. In addition, weak inter­molecular C—H⋯O hydrogen bonds also contribute to the crystal packing arrangement.

## Related literature

For applications of metallocene-based polymers, see: Caldwell *et al.* (2000 ▶); Peckham *et al.* (2001 ▶). For a similar structure, see: Dalir Kheirollahi *et al.* (2005 ▶).
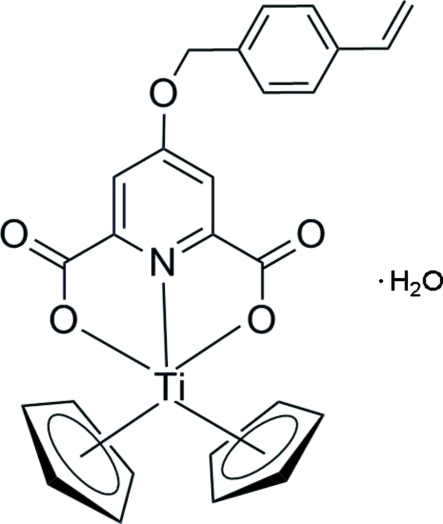


## Experimental

### 

#### Crystal data

[Ti(C_5_H_5_)_2_(C_16_H_11_NO_5_)]·H_2_O
*M* *_r_* = 493.35
Monoclinic, 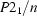

*a* = 7.1696 (7) Å
*b* = 13.7884 (13) Å
*c* = 22.419 (2) Å
β = 97.460 (1)°
*V* = 2197.6 (4) Å^3^
*Z* = 4
Mo *K*α radiation
μ = 0.44 mm^−1^
*T* = 153 K
0.32 × 0.28 × 0.23 mm

#### Data collection

Bruker APEXII CCD detector diffractometer
Absorption correction: multi-scan (*SADABS*; Bruker, 2001 ▶) *T*_min_ = 0.873, *T*_max_ = 0.907
13494 measured reflections
5269 independent reflections
3775 reflections with *I* > 2σ(*I*)
*R*_int_ = 0.098

#### Refinement

*R*[*F* ^2^ > 2σ(*F* ^2^)] = 0.045
*wR*(*F* ^2^) = 0.127
*S* = 1.03
5269 reflections
315 parameters
2 restraints
H atoms treated by a mixture of independent and constrained refinement
Δρ_max_ = 0.46 e Å^−3^
Δρ_min_ = −0.48 e Å^−3^

### 

Data collection: *APEX2* (Bruker, 2004 ▶); cell refinement: *SAINT* (Bruker, 2004 ▶); data reduction: *SAINT*; program(s) used to solve structure: *SHELXS97* (Sheldrick, 2008 ▶); program(s) used to refine structure: *SHELXL97* (Sheldrick, 2008 ▶); molecular graphics: *ORTEPIII* (Burnett & Johnson, 1996 ▶); software used to prepare material for publication: *SHELXL97*, *PLATON* (Spek, 2009 ▶) and *Mercury* (Macrae *et al.*, 2006 ▶).

## Supplementary Material

Crystal structure: contains datablocks global, I. DOI: 10.1107/S1600536810021148/zl2275sup1.cif

Structure factors: contains datablocks I. DOI: 10.1107/S1600536810021148/zl2275Isup2.hkl

Additional supplementary materials:  crystallographic information; 3D view; checkCIF report

## Figures and Tables

**Table 1:** Hydrogen-bond geometry (Å, °)

*D*—H⋯*A*	*D*—H	H⋯*A*	*D*⋯*A*	*D*—H⋯*A*
O6—H6*O*1⋯O2^i^	0.97 (4)	1.89 (4)	2.833 (2)	164 (3)
O6—H6*O*2⋯O2^ii^	1.08 (5)	1.79 (5)	2.847 (3)	165 (4)
C9—H9*A*⋯O6^iii^	0.99	2.59	3.464 (3)	148
C14—H14⋯O6^ii^	0.95	2.42	3.303 (3)	155
C17—H17⋯O6	1.00	2.59	3.227 (4)	121
C22—H22⋯O3^iv^	1.00	2.50	3.420 (3)	152
C23—H23⋯O4^v^	1.00	2.44	3.437 (3)	174

Symmetry codes: (i) 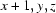; (ii) 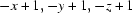; (iii) 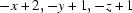; (iv) 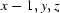; (v) 
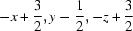
.

**Fig. 1.**
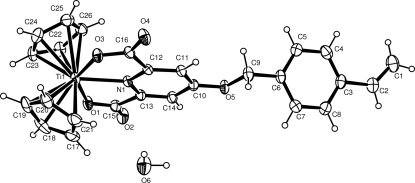
The molecular structure of the title compound, showing 50% probability displacement ellipsoids and the atomic numbering.

**Fig. 2.**
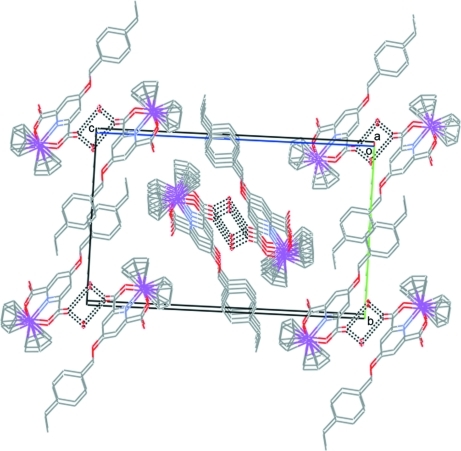
The packing in the title compound as viewed down the a-axis. Dashed lines indicate H-bonds but H atoms are omitted for clarity

## supplementary crystallographic information

### Comment

Metallocene-based polymers have attracted considerable attention and research interest in the areas of catalysts, photosensitizers, heat resisting materials, anticancer medicines and optical materials because of their excellent properties such as a high dielectric constant, high thermal stability and special rheological (Caldwell *et al.*, 2000; Peckham *et al.*, 2001). A number of pyridinecarboxylic acid titanocene-containing complexes have been synthesized (Dalir Kheirollahi *et al.*, 2005).

In the current contribution we would like to report the crystal structure of the title titanocene-containing complex, which also features a styrene functionality and might thus be polymerized or co-polymerized to yield metallocene-containing polymers. The compound crystallized as a monohydrate and the solvate water molecule forms strong O—H···O hydrogen bonds with the carboxylate O atoms of the Ti complex that play an important role in the connection of adjacent molecules (Figure 2). The water molecules are hydrogen bonded towards two symmetry dependent uncoordinated carboxylate oxygen atoms (O2) in neighboring molecules, with two water molecules bridging between two carboxylate O atoms so as to form a quadrilateral ring, thus connecting the complexes into hydrogen bonded dimers (Table 1, Figure 2). In addition, weak intermolecular C—H···O hydrogen bonds also contribute to the crystal packing arrangement (Table 1).

### Experimental

A solution of 4-(4-vinylbenzyloxy)pyridine-2,6-dicarboxylic acid (0.594 g, 2 mmol) and sodium carbonate (0.212 g, 2 mmol) in 20 ml water was added to a solution of bis(cyclopentadienyl) titanium dichloride (0.498 g, 2 mmol) in 30 ml water at 298 K. Then the mixture was stirred at 298 K for 10 min. After the reaction was completed, the solution was extracted with CHCl_3_ several times. The combined CHCl_3_ layers were dried over anhydrous Na_2_SO_4_. The product was obtained in 94.8% yield as a yellow powder after solvent removal under vacuum. The single crystals suitable for X-ray diffraction were obtained at ambient temperature by slow evaporation of a dichloromethane/hexane solution (5/1, v/v) over a period of several days. ^1^H NMR (500 MHz, CDCl_3_) δ (ppm): 7.73 (s, 2H), 7.47 (d 2H), 7.39 (d, 2H), 6.73 (q, 2H), 6.18 (s, 10H), 5.78 (d, 1H), 5.32 (s, 2H), 5.29 (d, 1H). IR (cm^-1^): 1652 (C=O), 1447 (Py), 993 (C=C), 825 (Cp). Elemental analysis calculated(%): C, 65.68; H, 4.42; N, 2.95. Found(%): C, 65.61; H, 4.49; N, 3.01.

### Refinement

Carbon bound H atoms were positioned geometrically and refined in the riding model approximation with C—H = 0.95, 0.99 and 1.00 Å, and with *U_iso_*(H) = 1.2 *U_eq_*(C). The water H-atoms were located in a difference Fourier map and were refined isotropically.

### Crystal data

**Table:**

[Ti(C_5_H_5_)_2_(C_16_H_11_NO_5_)]·H_2_O	*F*(000) = 1024
*M**_r_* = 493.35	*D*_x_ = 1.491 Mg m^−^^3^
Monoclinic, *P*2_1_/*n*	Mo *K*α radiation, λ = 0.71073 Å
Hall symbol: -P 2yn	Cell parameters from 4730 reflections
*a* = 7.1696 (7) Å	θ = 2.4–27.3°
*b* = 13.7884 (13) Å	µ = 0.44 mm^−^^1^
*c* = 22.419 (2) Å	*T* = 153 K
β = 97.460 (1)°	Block, colourless
*V* = 2197.6 (4) Å^3^	0.32 × 0.28 × 0.23 mm
*Z* = 4	

### Data collection

**Table:**

Bruker APEXII CCD detector diffractometer	5269 independent reflections
Radiation source: sealed tube	3775 reflections with *I* > 2σ(*I*)
graphite	*R*_int_ = 0.098
φ and ω scans	θ_max_ = 28.2°, θ_min_ = 1.7°
Absorption correction: multi-scan (*SADABS*; Bruker, 2001)	*h* = −9→9
*T*_min_ = 0.873, *T*_max_ = 0.907	*k* = −18→18
13494 measured reflections	*l* = −29→18

### Refinement

**Table:**

Refinement on *F*^2^	Primary atom site location: structure-invariant direct methods
Least-squares matrix: full	Secondary atom site location: difference Fourier map
*R*[*F*^2^ > 2σ(*F*^2^)] = 0.045	Hydrogen site location: mixed
*wR*(*F*^2^) = 0.127	H atoms treated by a mixture of independent and constrained refinement
*S* = 1.03	*w* = 1/[σ^2^(*F*_o_^2^) + (0.0558*P*)^2^] where *P* = (*F*_o_^2^ + 2*F*_c_^2^)/3
5269 reflections	(Δ/σ)_max_ = 0.001
315 parameters	Δρ_max_ = 0.46 e Å^−^^3^
2 restraints	Δρ_min_ = −0.48 e Å^−^^3^

### Special details

**Table:**

Geometry. All esds (except the esd in the dihedral angle between two l.s. planes) are estimated using the full covariance matrix. The cell esds are taken into account individually in the estimation of esds in distances, angles and torsion angles; correlations between esds in cell parameters are only used when they are defined by crystal symmetry. An approximate (isotropic) treatment of cell esds is used for estimating esds involving l.s. planes.
Refinement. Refinement of F^2^ against ALL reflections. The weighted R-factor wR and goodness of fit S are based on F^2^, conventional R-factors R are based on F, with F set to zero for negative F^2^. The threshold expression of F^2^ > 2sigma(F^2^) is used only for calculating R-factors(gt) etc. and is not relevant to the choice of reflections for refinement. R-factors based on F^2^ are statistically about twice as large as those based on F, and R- factors based on ALL data will be even larger.

### Fractional atomic coordinates and isotropic or equivalent isotropic displacement parameters (Å^2^)

**Table:**

	*x*	*y*	*z*	*U*_iso_*/*U*_eq_	
Ti1	0.69524 (4)	0.37174 (2)	0.697730 (16)	0.03351 (13)	
O1	0.4408 (2)	0.37378 (10)	0.63473 (7)	0.0461 (4)	
N1	0.6958 (2)	0.49830 (11)	0.63954 (7)	0.0314 (3)	
O4	1.1293 (2)	0.58056 (11)	0.70963 (7)	0.0501 (4)	
O5	0.68376 (18)	0.73586 (10)	0.52883 (6)	0.0433 (4)	
O3	0.95114 (17)	0.45046 (10)	0.72073 (6)	0.0388 (3)	
C11	0.8521 (3)	0.63889 (14)	0.60863 (8)	0.0334 (4)	
H11	0.9598	0.6797	0.6133	0.040*	
C16	0.9911 (2)	0.53006 (14)	0.69565 (9)	0.0340 (4)	
O2	0.2441 (2)	0.45197 (13)	0.56604 (7)	0.0581 (5)	
C12	0.8425 (2)	0.55837 (13)	0.64445 (8)	0.0306 (4)	
C10	0.6995 (3)	0.65870 (15)	0.56549 (9)	0.0354 (4)	
C6	0.7905 (3)	0.88739 (15)	0.49283 (10)	0.0405 (5)	
C15	0.3962 (3)	0.44286 (16)	0.59832 (9)	0.0406 (5)	
C14	0.5456 (3)	0.59511 (15)	0.55972 (9)	0.0371 (4)	
H14	0.4405	0.6064	0.5301	0.045*	
C13	0.5488 (2)	0.51691 (14)	0.59710 (8)	0.0338 (4)	
C3	0.7158 (3)	1.04503 (17)	0.41498 (10)	0.0455 (5)	
C9	0.8416 (3)	0.80338 (17)	0.53390 (10)	0.0476 (5)	
H9A	0.9547	0.7705	0.5227	0.057*	
H9B	0.8701	0.8264	0.5759	0.057*	
C2	0.6819 (3)	1.12760 (19)	0.37223 (12)	0.0559 (6)	
H2	0.6552	1.1110	0.3309	0.067*	
C22	0.4196 (3)	0.39584 (17)	0.74867 (11)	0.0468 (5)	
H22	0.2863	0.3958	0.7289	0.056*	
C23	0.5186 (3)	0.31475 (17)	0.77295 (10)	0.0484 (5)	
H23	0.4668	0.2478	0.7759	0.058*	
C4	0.7219 (3)	1.05683 (18)	0.47672 (11)	0.0524 (6)	
H4	0.7002	1.1190	0.4928	0.063*	
C1	0.6844 (3)	1.2187 (2)	0.38448 (14)	0.0651 (7)	
H1A	0.7102	1.2399	0.4250	0.078*	
H1B	0.6604	1.2649	0.3530	0.078*	
C26	0.5322 (3)	0.47828 (17)	0.76070 (10)	0.0475 (5)	
H26	0.4932	0.5468	0.7512	0.057*	
C19	0.7826 (4)	0.20477 (17)	0.71147 (13)	0.0595 (7)	
H19	0.7623	0.1633	0.7466	0.071*	
C7	0.7802 (3)	0.87685 (17)	0.43084 (11)	0.0514 (6)	
H7	0.7991	0.8146	0.4144	0.062*	
C21	0.9291 (4)	0.29268 (19)	0.64666 (12)	0.0599 (7)	
H21	1.0311	0.3259	0.6280	0.072*	
C25	0.6991 (3)	0.44875 (18)	0.79528 (10)	0.0497 (6)	
H25	0.7999	0.4928	0.8148	0.060*	
C8	0.7433 (3)	0.95444 (18)	0.39290 (10)	0.0539 (6)	
H8	0.7368	0.9451	0.3507	0.065*	
C24	0.6946 (3)	0.34904 (18)	0.80199 (10)	0.0503 (6)	
H24	0.7881	0.3097	0.8288	0.060*	
C5	0.7596 (3)	0.97820 (17)	0.51504 (10)	0.0477 (5)	
H5	0.7642	0.9872	0.5572	0.057*	
C20	0.9456 (3)	0.25547 (16)	0.70441 (12)	0.0551 (6)	
H20	1.0621	0.2573	0.7342	0.066*	
C18	0.6618 (4)	0.21283 (18)	0.65783 (15)	0.0706 (8)	
H18	0.5403	0.1772	0.6477	0.085*	
C17	0.7549 (5)	0.26670 (19)	0.61727 (13)	0.0701 (8)	
H17	0.7100	0.2768	0.5736	0.084*	
O6	0.8910 (3)	0.38835 (15)	0.50777 (9)	0.0714 (6)	
H6O1	1.011 (5)	0.400 (3)	0.5321 (15)	0.117 (12)*	
H6O2	0.864 (6)	0.452 (4)	0.4802 (18)	0.163 (17)*	

### Atomic displacement parameters (Å^2^)

**Table:**

	*U*^11^	*U*^22^	*U*^33^	*U*^12^	*U*^13^	*U*^23^
Ti1	0.0321 (2)	0.0242 (2)	0.0433 (2)	−0.00074 (13)	0.00121 (14)	0.00586 (14)
O1	0.0431 (8)	0.0374 (9)	0.0543 (9)	−0.0118 (6)	−0.0071 (7)	0.0121 (7)
N1	0.0303 (8)	0.0267 (8)	0.0361 (8)	−0.0017 (6)	0.0007 (6)	0.0031 (7)
O4	0.0366 (7)	0.0377 (9)	0.0708 (10)	−0.0081 (6)	−0.0129 (7)	0.0107 (8)
O5	0.0438 (8)	0.0369 (8)	0.0470 (8)	−0.0072 (6)	−0.0028 (6)	0.0157 (7)
O3	0.0323 (7)	0.0329 (8)	0.0495 (8)	0.0001 (6)	−0.0016 (6)	0.0107 (6)
C11	0.0328 (9)	0.0290 (10)	0.0381 (10)	−0.0031 (7)	0.0038 (8)	0.0017 (8)
C16	0.0310 (9)	0.0285 (10)	0.0418 (11)	0.0037 (8)	0.0022 (8)	0.0015 (8)
O2	0.0432 (9)	0.0624 (11)	0.0622 (10)	−0.0167 (7)	−0.0184 (7)	0.0183 (8)
C12	0.0296 (9)	0.0255 (9)	0.0364 (10)	0.0011 (7)	0.0028 (7)	−0.0009 (8)
C10	0.0393 (10)	0.0311 (10)	0.0358 (10)	−0.0015 (8)	0.0050 (8)	0.0051 (8)
C6	0.0356 (10)	0.0369 (12)	0.0490 (12)	−0.0066 (9)	0.0049 (9)	0.0083 (9)
C15	0.0382 (11)	0.0394 (12)	0.0421 (11)	−0.0070 (9)	−0.0026 (9)	0.0011 (9)
C14	0.0366 (10)	0.0359 (11)	0.0370 (10)	−0.0030 (8)	−0.0025 (8)	0.0052 (9)
C13	0.0323 (9)	0.0315 (11)	0.0361 (10)	−0.0031 (8)	−0.0008 (8)	0.0020 (8)
C3	0.0358 (11)	0.0494 (14)	0.0512 (13)	−0.0056 (9)	0.0046 (9)	0.0136 (11)
C9	0.0428 (11)	0.0417 (13)	0.0565 (13)	−0.0095 (9)	0.0001 (10)	0.0135 (11)
C2	0.0525 (13)	0.0530 (16)	0.0617 (15)	−0.0024 (11)	0.0058 (11)	0.0115 (12)
C22	0.0347 (11)	0.0464 (13)	0.0602 (14)	0.0028 (9)	0.0099 (10)	0.0091 (11)
C23	0.0474 (12)	0.0392 (12)	0.0614 (14)	−0.0036 (10)	0.0176 (10)	0.0147 (11)
C4	0.0506 (13)	0.0377 (13)	0.0711 (16)	0.0000 (10)	0.0153 (11)	−0.0018 (11)
C1	0.0554 (15)	0.0520 (17)	0.087 (2)	0.0061 (12)	0.0068 (13)	0.0186 (14)
C26	0.0511 (13)	0.0399 (13)	0.0536 (13)	0.0071 (10)	0.0147 (10)	0.0019 (10)
C19	0.0695 (16)	0.0265 (12)	0.0847 (19)	0.0092 (11)	0.0188 (14)	0.0112 (12)
C7	0.0635 (14)	0.0387 (13)	0.0526 (14)	−0.0015 (11)	0.0107 (11)	−0.0002 (10)
C21	0.0680 (16)	0.0430 (15)	0.0737 (18)	0.0082 (12)	0.0277 (14)	−0.0096 (13)
C25	0.0497 (13)	0.0539 (15)	0.0467 (13)	−0.0078 (11)	0.0106 (10)	−0.0068 (11)
C8	0.0642 (15)	0.0524 (16)	0.0451 (13)	−0.0051 (12)	0.0071 (11)	0.0061 (11)
C24	0.0451 (12)	0.0600 (16)	0.0462 (12)	0.0070 (11)	0.0067 (10)	0.0176 (11)
C5	0.0521 (13)	0.0463 (14)	0.0459 (12)	−0.0030 (10)	0.0111 (10)	0.0039 (10)
C20	0.0525 (13)	0.0373 (13)	0.0761 (17)	0.0127 (11)	0.0112 (12)	0.0029 (12)
C18	0.0653 (16)	0.0295 (13)	0.113 (2)	−0.0026 (11)	−0.0046 (16)	−0.0176 (14)
C17	0.109 (2)	0.0436 (15)	0.0559 (16)	0.0176 (15)	0.0047 (15)	−0.0135 (12)
O6	0.0559 (11)	0.0731 (14)	0.0798 (13)	−0.0169 (10)	−0.0114 (10)	0.0203 (11)

### Geometric parameters (Å, °)

**Table:**

Ti1—O3	2.1365 (13)	C9—H9A	0.9900
Ti1—O1	2.1573 (14)	C9—H9B	0.9900
Ti1—N1	2.1792 (15)	C2—C1	1.286 (3)
Ti1—C24	2.359 (2)	C2—H2	0.9500
Ti1—C18	2.367 (2)	C22—C23	1.397 (3)
Ti1—C23	2.371 (2)	C22—C26	1.400 (3)
Ti1—C17	2.395 (3)	C22—H22	1.0000
Ti1—C19	2.395 (2)	C23—C24	1.423 (3)
Ti1—C20	2.397 (2)	C23—H23	1.0000
Ti1—C21	2.410 (2)	C4—C5	1.388 (3)
Ti1—C25	2.428 (2)	C4—H4	0.9500
Ti1—C22	2.430 (2)	C1—H1A	0.9500
O1—C15	1.268 (2)	C1—H1B	0.9500
N1—C12	1.332 (2)	C26—C25	1.399 (3)
N1—C13	1.349 (2)	C26—H26	1.0000
O4—C16	1.218 (2)	C19—C20	1.388 (3)
O5—C10	1.340 (2)	C19—C18	1.392 (4)
O5—C9	1.458 (2)	C19—H19	1.0000
O3—C16	1.282 (2)	C7—C8	1.371 (3)
C11—C12	1.377 (3)	C7—H7	0.9500
C11—C10	1.390 (3)	C21—C17	1.382 (4)
C11—H11	0.9500	C21—C20	1.383 (4)
C16—C12	1.512 (2)	C21—H21	1.0000
O2—C15	1.235 (2)	C25—C24	1.384 (3)
C10—C14	1.402 (3)	C25—H25	1.0000
C6—C5	1.376 (3)	C8—H8	0.9500
C6—C7	1.390 (3)	C24—H24	1.0000
C6—C9	1.495 (3)	C5—H5	0.9500
C15—C13	1.499 (3)	C20—H20	1.0000
C14—C13	1.364 (3)	C18—C17	1.408 (4)
C14—H14	0.9500	C18—H18	1.0000
C3—C8	1.367 (3)	C17—H17	1.0000
C3—C4	1.389 (3)	O6—H6O1	0.97 (4)
C3—C2	1.488 (3)	O6—H6O2	1.08 (5)
			
O3—Ti1—O1	141.08 (5)	C10—C14—H14	120.4
O3—Ti1—N1	70.70 (5)	N1—C13—C14	122.19 (17)
O1—Ti1—N1	70.40 (5)	N1—C13—C15	111.25 (16)
O3—Ti1—C24	86.58 (7)	C14—C13—C15	126.54 (17)
O1—Ti1—C24	122.70 (7)	C8—C3—C4	118.7 (2)
N1—Ti1—C24	134.41 (8)	C8—C3—C2	119.0 (2)
O3—Ti1—C18	127.10 (8)	C4—C3—C2	122.3 (2)
O1—Ti1—C18	74.28 (8)	O5—C9—C6	108.73 (16)
N1—Ti1—C18	121.54 (9)	O5—C9—H9A	109.9
C24—Ti1—C18	103.80 (11)	C6—C9—H9A	109.9
O3—Ti1—C23	121.20 (7)	O5—C9—H9B	109.9
O1—Ti1—C23	89.18 (7)	C6—C9—H9B	109.9
N1—Ti1—C23	137.22 (7)	H9A—C9—H9B	108.3
C24—Ti1—C23	35.03 (8)	C1—C2—C3	127.9 (3)
C18—Ti1—C23	85.68 (10)	C1—C2—H2	116.1
O3—Ti1—C17	104.91 (9)	C3—C2—H2	116.1
O1—Ti1—C17	74.53 (9)	C23—C22—C26	108.9 (2)
N1—Ti1—C17	91.09 (9)	C23—C22—Ti1	70.76 (12)
C24—Ti1—C17	133.61 (9)	C26—C22—Ti1	73.61 (12)
C18—Ti1—C17	34.39 (10)	C23—C22—H22	125.5
C23—Ti1—C17	119.97 (10)	C26—C22—H22	125.5
O3—Ti1—C19	104.79 (7)	Ti1—C22—H22	125.5
O1—Ti1—C19	106.35 (8)	C22—C23—C24	106.7 (2)
N1—Ti1—C19	145.69 (8)	C22—C23—Ti1	75.45 (13)
C24—Ti1—C19	77.24 (9)	C24—C23—Ti1	72.03 (12)
C18—Ti1—C19	33.99 (9)	C22—C23—H23	126.2
C23—Ti1—C19	75.21 (9)	C24—C23—H23	126.2
C17—Ti1—C19	56.38 (9)	Ti1—C23—H23	126.2
O3—Ti1—C20	73.27 (7)	C5—C4—C3	120.3 (2)
O1—Ti1—C20	127.76 (8)	C5—C4—H4	119.9
N1—Ti1—C20	120.90 (8)	C3—C4—H4	119.9
C24—Ti1—C20	86.97 (9)	C2—C1—H1A	120.0
C18—Ti1—C20	56.14 (9)	C2—C1—H1B	120.0
C23—Ti1—C20	101.46 (8)	H1A—C1—H1B	120.0
C17—Ti1—C20	55.74 (9)	C25—C26—C22	107.5 (2)
C19—Ti1—C20	33.69 (8)	C25—C26—Ti1	72.89 (13)
O3—Ti1—C21	73.49 (8)	C22—C26—Ti1	72.97 (13)
O1—Ti1—C21	106.03 (8)	C25—C26—H26	126.0
N1—Ti1—C21	91.28 (8)	C22—C26—H26	126.0
C24—Ti1—C21	120.12 (9)	Ti1—C26—H26	126.0
C18—Ti1—C21	56.25 (10)	C20—C19—C18	107.4 (2)
C23—Ti1—C21	131.06 (9)	C20—C19—Ti1	73.22 (13)
C17—Ti1—C21	33.42 (9)	C18—C19—Ti1	71.90 (13)
C19—Ti1—C21	55.95 (9)	C20—C19—H19	126.1
C20—Ti1—C21	33.45 (8)	C18—C19—H19	126.1
O3—Ti1—C25	69.60 (7)	Ti1—C19—H19	126.1
O1—Ti1—C25	119.59 (7)	C8—C7—C6	121.3 (2)
N1—Ti1—C25	100.85 (7)	C8—C7—H7	119.3
C24—Ti1—C25	33.57 (8)	C6—C7—H7	119.3
C18—Ti1—C25	137.22 (10)	C17—C21—C20	108.2 (2)
C23—Ti1—C25	56.56 (8)	C17—C21—Ti1	72.68 (15)
C17—Ti1—C25	163.88 (9)	C20—C21—Ti1	72.74 (13)
C19—Ti1—C25	109.41 (9)	C17—C21—H21	125.7
C20—Ti1—C25	108.38 (9)	C20—C21—H21	125.7
C21—Ti1—C25	134.30 (9)	Ti1—C21—H21	125.7
O3—Ti1—C22	122.84 (7)	C24—C25—C26	108.6 (2)
O1—Ti1—C22	68.64 (7)	C24—C25—Ti1	70.47 (13)
N1—Ti1—C22	103.68 (7)	C26—C25—Ti1	73.69 (13)
C24—Ti1—C22	56.37 (8)	C24—C25—H25	125.6
C18—Ti1—C22	104.80 (10)	C26—C25—H25	125.6
C23—Ti1—C22	33.79 (7)	Ti1—C25—H25	125.6
C17—Ti1—C22	132.24 (10)	C3—C8—C7	120.9 (2)
C19—Ti1—C22	106.62 (9)	C3—C8—H8	119.5
C20—Ti1—C22	135.25 (8)	C7—C8—H8	119.5
C21—Ti1—C22	160.63 (9)	C25—C24—C23	108.19 (19)
C25—Ti1—C22	55.38 (8)	C25—C24—Ti1	75.96 (13)
C15—O1—Ti1	123.54 (12)	C23—C24—Ti1	72.94 (12)
C12—N1—C13	118.42 (16)	C25—C24—H24	125.5
C12—N1—Ti1	120.74 (11)	C23—C24—H24	125.5
C13—N1—Ti1	120.83 (12)	Ti1—C24—H24	125.5
C10—O5—C9	117.14 (15)	C6—C5—C4	121.0 (2)
C16—O3—Ti1	124.24 (11)	C6—C5—H5	119.5
C12—C11—C10	118.02 (17)	C4—C5—H5	119.5
C12—C11—H11	121.0	C21—C20—C19	108.8 (2)
C10—C11—H11	121.0	C21—C20—Ti1	73.81 (13)
O4—C16—O3	126.64 (17)	C19—C20—Ti1	73.10 (13)
O4—C16—C12	121.14 (18)	C21—C20—H20	125.4
O3—C16—C12	112.22 (16)	C19—C20—H20	125.4
N1—C12—C11	123.48 (16)	Ti1—C20—H20	125.4
N1—C12—C16	111.92 (16)	C19—C18—C17	107.8 (2)
C11—C12—C16	124.54 (16)	C19—C18—Ti1	74.11 (14)
O5—C10—C11	125.36 (17)	C17—C18—Ti1	73.88 (15)
O5—C10—C14	115.93 (16)	C19—C18—H18	125.7
C11—C10—C14	118.70 (18)	C17—C18—H18	125.7
C5—C6—C7	117.8 (2)	Ti1—C18—H18	125.7
C5—C6—C9	121.3 (2)	C21—C17—C18	107.7 (2)
C7—C6—C9	120.8 (2)	C21—C17—Ti1	73.90 (14)
O2—C15—O1	125.71 (19)	C18—C17—Ti1	71.73 (15)
O2—C15—C13	120.75 (18)	C21—C17—H17	125.9
O1—C15—C13	113.55 (16)	C18—C17—H17	125.9
C13—C14—C10	119.17 (17)	Ti1—C17—H17	125.9
C13—C14—H14	120.4	H6O1—O6—H6O2	106 (3)
			
O3—Ti1—O1—C15	−7.6 (2)	N1—Ti1—C19—C18	−59.1 (2)
N1—Ti1—O1—C15	−5.47 (16)	C24—Ti1—C19—C18	140.65 (19)
C24—Ti1—O1—C15	125.39 (18)	C23—Ti1—C19—C18	104.61 (18)
C18—Ti1—O1—C15	−138.1 (2)	C17—Ti1—C19—C18	−38.14 (17)
C23—Ti1—O1—C15	136.18 (18)	C20—Ti1—C19—C18	−115.3 (2)
C17—Ti1—O1—C15	−102.33 (19)	C21—Ti1—C19—C18	−78.64 (18)
C19—Ti1—O1—C15	−149.50 (18)	C25—Ti1—C19—C18	150.40 (17)
C20—Ti1—O1—C15	−119.92 (18)	C22—Ti1—C19—C18	91.96 (18)
C21—Ti1—O1—C15	−91.02 (19)	C5—C6—C7—C8	1.0 (3)
C25—Ti1—O1—C15	86.12 (19)	C9—C6—C7—C8	−176.5 (2)
C22—Ti1—O1—C15	108.66 (18)	O3—Ti1—C21—C17	−159.45 (18)
O3—Ti1—N1—C12	1.13 (13)	O1—Ti1—C21—C17	−20.07 (18)
O1—Ti1—N1—C12	−177.44 (15)	N1—Ti1—C21—C17	−90.03 (17)
C24—Ti1—N1—C12	65.56 (17)	C24—Ti1—C21—C17	124.66 (17)
C18—Ti1—N1—C12	−121.21 (15)	C18—Ti1—C21—C17	37.87 (16)
C23—Ti1—N1—C12	116.57 (15)	C23—Ti1—C21—C17	83.3 (2)
C17—Ti1—N1—C12	−104.30 (16)	C19—Ti1—C21—C17	79.10 (18)
C19—Ti1—N1—C12	−86.97 (19)	C20—Ti1—C21—C17	116.1 (2)
C20—Ti1—N1—C12	−54.46 (17)	C25—Ti1—C21—C17	163.41 (16)
C21—Ti1—N1—C12	−70.87 (15)	C22—Ti1—C21—C17	50.9 (3)
C25—Ti1—N1—C12	64.82 (15)	O3—Ti1—C21—C20	84.48 (15)
C22—Ti1—N1—C12	121.54 (15)	O1—Ti1—C21—C20	−136.14 (15)
O3—Ti1—N1—C13	−179.87 (15)	N1—Ti1—C21—C20	153.90 (16)
O1—Ti1—N1—C13	1.56 (14)	C24—Ti1—C21—C20	8.59 (19)
C24—Ti1—N1—C13	−115.44 (15)	C18—Ti1—C21—C20	−78.20 (17)
C18—Ti1—N1—C13	57.79 (17)	C23—Ti1—C21—C20	−32.8 (2)
C23—Ti1—N1—C13	−64.42 (18)	C17—Ti1—C21—C20	−116.1 (2)
C17—Ti1—N1—C13	74.70 (16)	C19—Ti1—C21—C20	−36.97 (15)
C19—Ti1—N1—C13	92.04 (19)	C25—Ti1—C21—C20	47.3 (2)
C20—Ti1—N1—C13	124.55 (15)	C22—Ti1—C21—C20	−65.1 (3)
C21—Ti1—N1—C13	108.13 (16)	C22—C26—C25—C24	3.0 (3)
C25—Ti1—N1—C13	−116.18 (15)	Ti1—C26—C25—C24	−62.23 (16)
C22—Ti1—N1—C13	−59.46 (16)	C22—C26—C25—Ti1	65.26 (16)
O1—Ti1—O3—C16	4.2 (2)	O3—Ti1—C25—C24	−116.45 (14)
N1—Ti1—O3—C16	2.05 (15)	O1—Ti1—C25—C24	105.53 (14)
C24—Ti1—O3—C16	−137.75 (16)	N1—Ti1—C25—C24	179.04 (13)
C18—Ti1—O3—C16	117.51 (18)	C18—Ti1—C25—C24	6.6 (2)
C23—Ti1—O3—C16	−132.14 (16)	C23—Ti1—C25—C24	38.78 (13)
C17—Ti1—O3—C16	87.90 (16)	C17—Ti1—C25—C24	−43.8 (4)
C19—Ti1—O3—C16	146.41 (16)	C19—Ti1—C25—C24	−17.37 (15)
C20—Ti1—O3—C16	134.39 (17)	C20—Ti1—C25—C24	−53.02 (15)
C21—Ti1—O3—C16	99.43 (17)	C21—Ti1—C25—C24	−78.31 (18)
C25—Ti1—O3—C16	−108.02 (17)	C22—Ti1—C25—C24	79.82 (15)
C22—Ti1—O3—C16	−92.09 (16)	O3—Ti1—C25—C26	126.40 (15)
Ti1—O3—C16—O4	175.14 (16)	O1—Ti1—C25—C26	−11.62 (16)
Ti1—O3—C16—C12	−4.3 (2)	N1—Ti1—C25—C26	61.89 (14)
C13—N1—C12—C11	0.3 (3)	C24—Ti1—C25—C26	−117.1 (2)
Ti1—N1—C12—C11	179.30 (14)	C18—Ti1—C25—C26	−110.53 (17)
C13—N1—C12—C16	177.54 (16)	C23—Ti1—C25—C26	−78.36 (14)
Ti1—N1—C12—C16	−3.4 (2)	C17—Ti1—C25—C26	−160.9 (3)
C10—C11—C12—N1	0.6 (3)	C19—Ti1—C25—C26	−134.52 (14)
C10—C11—C12—C16	−176.32 (17)	C20—Ti1—C25—C26	−170.17 (14)
O4—C16—C12—N1	−174.76 (18)	C21—Ti1—C25—C26	164.54 (14)
O3—C16—C12—N1	4.7 (2)	C22—Ti1—C25—C26	−37.33 (13)
O4—C16—C12—C11	2.5 (3)	C4—C3—C8—C7	−1.3 (3)
O3—C16—C12—C11	−178.03 (18)	C2—C3—C8—C7	178.2 (2)
C9—O5—C10—C11	1.3 (3)	C6—C7—C8—C3	0.1 (4)
C9—O5—C10—C14	179.90 (18)	C26—C25—C24—C23	−2.1 (3)
C12—C11—C10—O5	177.28 (19)	Ti1—C25—C24—C23	−66.39 (16)
C12—C11—C10—C14	−1.3 (3)	C26—C25—C24—Ti1	64.30 (16)
Ti1—O1—C15—O2	−171.98 (17)	C22—C23—C24—C25	0.3 (3)
Ti1—O1—C15—C13	7.9 (3)	Ti1—C23—C24—C25	68.41 (16)
O5—C10—C14—C13	−177.60 (19)	C22—C23—C24—Ti1	−68.08 (16)
C11—C10—C14—C13	1.1 (3)	O3—Ti1—C24—C25	57.21 (13)
C12—N1—C13—C14	−0.5 (3)	O1—Ti1—C24—C25	−95.39 (14)
Ti1—N1—C13—C14	−179.50 (15)	N1—Ti1—C24—C25	−1.32 (18)
C12—N1—C13—C15	−179.46 (16)	C18—Ti1—C24—C25	−175.38 (14)
Ti1—N1—C13—C15	1.5 (2)	C23—Ti1—C24—C25	−114.42 (19)
C10—C14—C13—N1	−0.2 (3)	C17—Ti1—C24—C25	164.61 (16)
C10—C14—C13—C15	178.62 (19)	C19—Ti1—C24—C25	163.22 (15)
O2—C15—C13—N1	174.18 (19)	C20—Ti1—C24—C25	130.61 (14)
O1—C15—C13—N1	−5.7 (3)	C21—Ti1—C24—C25	125.88 (14)
O2—C15—C13—C14	−4.8 (3)	C22—Ti1—C24—C25	−76.60 (15)
O1—C15—C13—C14	175.3 (2)	O3—Ti1—C24—C23	171.63 (14)
C10—O5—C9—C6	−175.81 (18)	O1—Ti1—C24—C23	19.03 (17)
C5—C6—C9—O5	110.4 (2)	N1—Ti1—C24—C23	113.10 (14)
C7—C6—C9—O5	−72.2 (3)	C18—Ti1—C24—C23	−60.96 (15)
C8—C3—C2—C1	−167.4 (3)	C17—Ti1—C24—C23	−80.97 (18)
C4—C3—C2—C1	12.1 (4)	C19—Ti1—C24—C23	−82.37 (15)
O3—Ti1—C22—C23	−98.28 (15)	C20—Ti1—C24—C23	−114.97 (15)
O1—Ti1—C22—C23	123.83 (15)	C21—Ti1—C24—C23	−119.70 (14)
N1—Ti1—C22—C23	−173.93 (14)	C25—Ti1—C24—C23	114.42 (19)
C24—Ti1—C22—C23	−39.25 (14)	C22—Ti1—C24—C23	37.81 (13)
C18—Ti1—C22—C23	57.67 (16)	C7—C6—C5—C4	−0.9 (3)
C17—Ti1—C22—C23	81.74 (18)	C9—C6—C5—C4	176.62 (19)
C19—Ti1—C22—C23	22.37 (16)	C3—C4—C5—C6	−0.3 (3)
C20—Ti1—C22—C23	1.2 (2)	C17—C21—C20—C19	0.7 (3)
C21—Ti1—C22—C23	46.4 (3)	Ti1—C21—C20—C19	65.25 (17)
C25—Ti1—C22—C23	−80.06 (15)	C17—C21—C20—Ti1	−64.51 (17)
O3—Ti1—C22—C26	19.10 (15)	C18—C19—C20—C21	−1.5 (3)
O1—Ti1—C22—C26	−118.78 (14)	Ti1—C19—C20—C21	−65.71 (17)
N1—Ti1—C22—C26	−56.55 (14)	C18—C19—C20—Ti1	64.22 (17)
C24—Ti1—C22—C26	78.13 (15)	O3—Ti1—C20—C21	−85.23 (16)
C18—Ti1—C22—C26	175.05 (14)	O1—Ti1—C20—C21	57.39 (18)
C23—Ti1—C22—C26	117.4 (2)	N1—Ti1—C20—C21	−30.84 (18)
C17—Ti1—C22—C26	−160.88 (14)	C24—Ti1—C20—C21	−172.57 (16)
C19—Ti1—C22—C26	139.76 (14)	C18—Ti1—C20—C21	78.58 (18)
C20—Ti1—C22—C26	118.57 (15)	C23—Ti1—C20—C21	155.36 (16)
C21—Ti1—C22—C26	163.8 (2)	C17—Ti1—C20—C21	36.77 (16)
C25—Ti1—C22—C26	37.32 (13)	C19—Ti1—C20—C21	116.1 (2)
C26—C22—C23—C24	1.5 (3)	C25—Ti1—C20—C21	−146.32 (16)
Ti1—C22—C23—C24	65.74 (15)	C22—Ti1—C20—C21	154.69 (15)
C26—C22—C23—Ti1	−64.19 (16)	O3—Ti1—C20—C19	158.72 (17)
O3—Ti1—C23—C22	103.60 (14)	O1—Ti1—C20—C19	−58.66 (19)
O1—Ti1—C23—C22	−50.69 (14)	N1—Ti1—C20—C19	−146.89 (15)
N1—Ti1—C23—C22	8.7 (2)	C24—Ti1—C20—C19	71.38 (17)
C24—Ti1—C23—C22	113.4 (2)	C18—Ti1—C20—C19	−37.47 (16)
C18—Ti1—C23—C22	−124.99 (16)	C23—Ti1—C20—C19	39.31 (18)
C17—Ti1—C23—C22	−122.25 (16)	C17—Ti1—C20—C19	−79.28 (19)
C19—Ti1—C23—C22	−157.84 (16)	C21—Ti1—C20—C19	−116.1 (2)
C20—Ti1—C23—C22	−179.15 (15)	C25—Ti1—C20—C19	97.63 (17)
C21—Ti1—C23—C22	−161.41 (14)	C22—Ti1—C20—C19	38.6 (2)
C25—Ti1—C23—C22	76.28 (15)	C20—C19—C18—C17	1.7 (3)
O3—Ti1—C23—C24	−9.79 (16)	Ti1—C19—C18—C17	66.75 (18)
O1—Ti1—C23—C24	−164.07 (14)	C20—C19—C18—Ti1	−65.09 (17)
N1—Ti1—C23—C24	−104.69 (15)	O3—Ti1—C18—C19	56.7 (2)
C18—Ti1—C23—C24	121.63 (15)	O1—Ti1—C18—C19	−160.13 (19)
C17—Ti1—C23—C24	124.36 (15)	N1—Ti1—C18—C19	145.42 (15)
C19—Ti1—C23—C24	88.78 (15)	C24—Ti1—C18—C19	−39.55 (18)
C20—Ti1—C23—C24	67.47 (15)	C23—Ti1—C18—C19	−69.77 (17)
C21—Ti1—C23—C24	85.21 (17)	C17—Ti1—C18—C19	114.4 (2)
C25—Ti1—C23—C24	−37.11 (13)	C20—Ti1—C18—C19	37.13 (16)
C22—Ti1—C23—C24	−113.4 (2)	C21—Ti1—C18—C19	77.66 (17)
C8—C3—C4—C5	1.4 (3)	C25—Ti1—C18—C19	−43.3 (2)
C2—C3—C4—C5	−178.0 (2)	C22—Ti1—C18—C19	−97.89 (17)
C23—C22—C26—C25	−2.8 (3)	O3—Ti1—C18—C17	−57.7 (2)
Ti1—C22—C26—C25	−65.21 (15)	O1—Ti1—C18—C17	85.44 (17)
C23—C22—C26—Ti1	62.38 (16)	N1—Ti1—C18—C17	31.00 (19)
O3—Ti1—C26—C25	−48.98 (14)	C24—Ti1—C18—C17	−153.98 (16)
O1—Ti1—C26—C25	169.90 (14)	C23—Ti1—C18—C17	175.80 (17)
N1—Ti1—C26—C25	−119.49 (14)	C19—Ti1—C18—C17	−114.4 (2)
C24—Ti1—C26—C25	36.31 (13)	C20—Ti1—C18—C17	−77.30 (17)
C18—Ti1—C26—C25	107.89 (18)	C21—Ti1—C18—C17	−36.77 (16)
C23—Ti1—C26—C25	78.72 (14)	C25—Ti1—C18—C17	−157.74 (16)
C17—Ti1—C26—C25	163.7 (3)	C22—Ti1—C18—C17	147.69 (16)
C19—Ti1—C26—C25	61.25 (17)	C20—C21—C17—C18	0.3 (3)
C20—Ti1—C26—C25	14.9 (2)	Ti1—C21—C17—C18	−64.25 (18)
C21—Ti1—C26—C25	−44.9 (4)	C20—C21—C17—Ti1	64.55 (17)
C22—Ti1—C26—C25	115.06 (19)	C19—C18—C17—C21	−1.2 (3)
O3—Ti1—C26—C22	−164.04 (13)	Ti1—C18—C17—C21	65.69 (18)
O1—Ti1—C26—C22	54.84 (13)	C19—C18—C17—Ti1	−66.91 (17)
N1—Ti1—C26—C22	125.45 (13)	O3—Ti1—C17—C21	20.38 (18)
C24—Ti1—C26—C22	−78.75 (14)	O1—Ti1—C17—C21	159.99 (18)
C18—Ti1—C26—C22	−7.2 (2)	N1—Ti1—C17—C21	90.68 (17)
C23—Ti1—C26—C22	−36.34 (13)	C24—Ti1—C17—C21	−79.3 (2)
C17—Ti1—C26—C22	48.7 (3)	C18—Ti1—C17—C21	−115.4 (2)
C19—Ti1—C26—C22	−53.81 (17)	C23—Ti1—C17—C21	−120.19 (18)
C20—Ti1—C26—C22	−100.11 (16)	C19—Ti1—C17—C21	−77.68 (17)
C21—Ti1—C26—C22	−160.0 (3)	C20—Ti1—C17—C21	−36.81 (15)
C25—Ti1—C26—C22	−115.06 (19)	C25—Ti1—C17—C21	−47.4 (4)
O3—Ti1—C19—C20	−21.07 (17)	C22—Ti1—C17—C21	−159.64 (15)
O1—Ti1—C19—C20	135.28 (16)	O3—Ti1—C17—C18	135.74 (16)
N1—Ti1—C19—C20	56.2 (2)	O1—Ti1—C17—C18	−84.65 (17)
C24—Ti1—C19—C20	−104.00 (17)	N1—Ti1—C17—C18	−153.96 (16)
C18—Ti1—C19—C20	115.3 (2)	C24—Ti1—C17—C18	36.0 (2)
C23—Ti1—C19—C20	−140.05 (18)	C23—Ti1—C17—C18	−4.8 (2)
C17—Ti1—C19—C20	77.21 (18)	C19—Ti1—C17—C18	37.68 (15)
C21—Ti1—C19—C20	36.71 (15)	C20—Ti1—C17—C18	78.55 (17)
C25—Ti1—C19—C20	−94.26 (17)	C21—Ti1—C17—C18	115.4 (2)
C22—Ti1—C19—C20	−152.69 (16)	C25—Ti1—C17—C18	67.9 (4)
O3—Ti1—C19—C18	−136.42 (17)	C22—Ti1—C17—C18	−44.3 (2)
O1—Ti1—C19—C18	19.93 (19)		

### Hydrogen-bond geometry (Å, °)

**Table:**

*D*—H···*A*	*D*—H	H···*A*	*D*···*A*	*D*—H···*A*
O6—H6O1···O2^i^	0.97 (4)	1.89 (4)	2.833 (2)	164 (3)
O6—H6O2···O2^ii^	1.08 (5)	1.79 (5)	2.847 (3)	165 (4)
C9—H9A···O6^iii^	0.99	2.59	3.464 (3)	148
C14—H14···O6^ii^	0.95	2.42	3.303 (3)	155
C17—H17···O6	1.00	2.59	3.227 (4)	121
C22—H22···O3^iv^	1.00	2.50	3.420 (3)	152
C23—H23···O4^v^	1.00	2.44	3.437 (3)	174

Symmetry codes: (i) *x*+1, *y*, *z*; (ii) −*x*+1, −*y*+1, −*z*+1; (iii) −*x*+2, −*y*+1, −*z*+1; (iv) *x*−1, *y*, *z*; (v) −*x*+3/2, *y*−1/2, −*z*+3/2.
